# Ripening of Sudanese Braided (Muddaffara) Cheese Manufactured from Raw or Pasteurized Milk: Effect of Heat Treatment and Salt Concentration on the Physicochemical Properties

**DOI:** 10.1155/2014/698263

**Published:** 2014-05-22

**Authors:** Mohamed O. E. Altahir, Elgasim A. Elgasim, Isam A. Mohamed Ahmed

**Affiliations:** ^1^Department of Food Science and Technology, Faculty of Agriculture, Omdurman Islamic University, 14415 Omdurman, Sudan; ^2^Department of Food Science and Technology, Faculty of Agriculture, University of Khartoum, Shambat, 13314 Khartoum North, Sudan

## Abstract

The objective of the study was to investigate the interactive effect of heat treatment (raw or pasteurized milk), ripening in salted whey (SW) and storage period for up to 3 months on the physicochemical properties of Sudanese braided cheese (SBC). Braided cheeses were manufactured from raw (BCRM) and pasteurized (BCPM) milk and ripened in SW (0%, 5%, and 10% salt) for up to 90 days. All the treatments significantly (*P* ≤ 0.05) affected the physicochemical characteristics of SBC. The total solid, protein, and fat contents of BCRM or BCPM decreased (*P* ≤ 0.05), whereas their TA, SN, and salt contents increased significantly (*P* ≤ 0.05) as storage period and the salt level of the whey were elevated. Both FRI and SRI of BCRM and BCPM increased with the increase in storage period and the salt level of the whey. For SN, FRI, SRI, pH, and moisture contents the magnitude of the change was more pronounced in BCRM than in BCPM, while for protein, fat, salt, and TS contents, the opposite was true; that is, the magnitude of the change was more pronounced in BCPM than in BCRM. Further studies are required to standardize muddaffara cheese manufacturing procedure particularly in rural areas.

## 1. Introduction

Cheese is a dairy product that has played a key role in human nutrition for centuries. It is the most popular dairy product in Sudan. Among cheese varieties available on the market, braided cheese is gaining popularity and became second to the white soft cheese (Gibna Bayda) as far as per capita consumption is concerned. The ripening process of cheese is very complex and involves microbiological [1] and biochemical [2–5] changes to the curd resulting in the flavor and texture characteristic of the particular variety. The ripening process of cheese is influenced by the ripening conditions like salt concentration and ripening duration. Salting was probably the most common and reliable traditional method used in combination with lactic acid fermentation for the production and preservation of cheese [6]. It was achieved by adding salt to milk before fermentation, sprinkling of dry salt on the surface of the cheese after moulding, and/or dipping the cheese in brine (pickling). The concentration and distribution of salt in cheese mass are important parameters affecting its quality and acceptability [7]. The low salt cooked cheese is more acceptable and has a low microbial count than low salt uncooked cheese [8]. Sodium chloride influences cheese ripening principally through its effect on water activity, control various enzyme activities in cheese, synergies of the curd, and physical changes in proteins which influence cheese texture and protein solubility [9]. Salt has three major functions in cheese: it acts as preservative, contributes directly to the flavor, and is a source of dietary sodium [10]. On the other hand, heat treatment of milk results in low bacterial load with an extended shelf life, permitting milk to be transported long distances or held for long periods before cheese manufacture. However, the cheese making characteristics of heated milks differ markedly from those typically of unheated milk [11]. It is well known that heat treatment of milk results in the denaturation of the whey proteins and interaction between the denatured whey proteins and the k-casein at the micelle surface [12]. The interaction involves thiol-disulphide bond interchange reactions between the free thiols of the denatured whey proteins (particularly *β*-lactoglobulin) and the disulphide bonds of k-casein. As the disulphide bonds of k-casein are found in the para-k-casein region, the denatured whey proteins will remain with the para-k-casein (micelles) after hydrolysis of k-casein by chymosin. Therefore, the heat treatment of milk prior to cheese-making is of considerable interest, as it appears to have the potential to substantially increase yield by incorporating the whey proteins in the cheese curd [13].

One typical cheese variety now being produced in the Sudan is the semihard braided cheese known locally as muddaffara or majdula cheese, which is a type of braided cheese in a special way to form a product of better keeping quality than the well-known white cheese. Braided cheese production in the Sudan is a small business, and the different producers adopt standard methods. About 7500 tons of braided cheese is manufactured in Sudan each year and this cheese is sold in the local markets [14]. However, research dealing with the physiochemical properties of Sudanese braided cheese made from raw cow milk is very meager. Moreover, there is no study on the Sudanese braided cheese as affected by use of pasteurized cow milk and different level of low salt on physicochemical quality during ripening. It is necessary to obtain best specifications for braided cheese and best quality attributes and to extend the shelf life of Sudanese braided cheese. The aim of this study was to investigate the combined effect of heat and salt concentration on physicochemical properties of Sudanese braided cheese during storage periods.

## 2. Materials and Methods

### 2.1. Materials

Fresh cow's milk was obtained from University of Khartoum Farm. Rennet (Chr. Hansen's, Denmark) and CaCl_2_ were products of Sigma. NaCl and Black cumin (*Nigella sativa*) were obtained from the local market. All chemicals and reagents used were of technically recommended analytical grade.

### 2.2. Cheese Manufacture

Braided cheese was manufactured as described previously [15]. Briefly, the cheese made from the fresh cow milk (100 L) was divided into two equal portions. One was used as raw milk without pasteurization and the other was pasteurized. Both milks were warmed to 40°C, and then starter culture with a combination of 1 : 1* Lactobacillus bulgaricus* and* Streptococcus thermophilus* (0.02%), rennet (1 tablet/50 kg milk), and CaCl_2_ were added. After complete coagulation (about 45 min), the curd (13.8–14.5 kg) was cut or broken to small parts and incubated until the required acidity (0.49–0.67 lactic acid %) for kneading was reached. The curd was put on a wooden table and left for 5 min to drain off the remaining whey. The curd was then cooked in a little whey at 70–80°C for five minutes. Thereafter, black cumin (*Nigella sativa*) was added (0.5%) to the hot paste, then cut into small pieces, and flattened like a circle shape. The curds formed were braided, put into plastic container containing salted whey (0.5 L) in three salt levels (0, 5, and 10%), and stored for up to 90 days at 5 ± 2°C.

### 2.3. pH Measurement

The pH was determined in 10% suspension of cheese in water according to AOAC official method [16]. Cheese (10 g) was thoroughly blended with 90 mL distilled water using a mortar and pestle. The pH of the resulting slurry was then measured potentiometrically using a pH meter (Model L15-1260/7, Pusl, Munchen, Germany).

### 2.4. Titratable Acidity

The titratable acidity (TA) of cheese was determined according to the AOAC method [16]. Briefly, 10 g of grated cheese or 10 mL of milk was weighed into a conical flask, and distilled water at 40°C was added until the volume in the flask reached 105 mL. The sample was then vigorously agitated and filtered. Twenty-five milliliters of the filtrate (Whatman 42 filter paper) was pipetted into a porcelain dish and titrated against 0.1 N NaOH using phenolphthalein as indicator of the end point. The acidity was calculated as percentage of lactic acid.

### 2.5. Moisture Content and Total Solids

The moisture content (MC) and total solids (TS) in cheese and milk were determined by drying samples in an oven at 103 ± 2°C until a constant weight was obtained (method number 926.08 AOAC [16]).

### 2.6. Total Content in Protein

Total nitrogen (TN) was determined according to the official methods [17]. Two grams of grated cheese in a beaker was mixed with few mL of distilled water and an equal amount of nitrogen-free sulphuric acid and then stirred gently. Thereafter, more water and sulphuric acid were added and stirred gently rubbing the sides of the beaker with glass rod in order to dislodge any curd adhered. The contents were transferred to Kjeldahl flask and sulphuric acid was added till the volume reached 25 mL. Then, 2 g of pure potassium sulphate and 0.3 g of copper sulphate were added. The flask was heated over a small flame and heating was continued for half an hour after the liquid became clear and then 50–60 mL of distilled water was added. Steam distillation was carried out with 50% NaOH and the distillate was collected in 25 mL of boric acid solution with added indicator (methyl red and bromocresol green). The content in the beaker was titrated against 0.02 N H_2_SO_4_ and the total content in protein was obtained by multiplying the amount of total nitrogen with the factor 6.38.

### 2.7. Soluble Nitrogen

The soluble nitrogen (SN) in cheese aqueous extract was determined by the Kjeldahl method according to AOAC methods [17]. Two grams of cheese was weighed into a small beaker, 25 mL of warm distilled water was added, and contents were ground gently and transferred into a 100 mL graduated flask. The volume was made up to 100 mL with distilled water. The contents were mixed well, filtered, and 5 mL of the filtrate was transferred with a pipette into a Kjeldahl flask. The procedure for measuring total nitrogen was then used to estimate SN in cheese.

### 2.8. Fat Content

The fat content was determined by the Gerber method as described by AOAC [17]. Briefly, 10 mL sulphuric acid (density 1.815 gm/mL) was poured into clean dry Gerber tubes, and then 10.94 mL of milk was added. For cheese testing, 3 g of minced cheese was added to the sulphuric acid as above, and then 1 mL of amyl alcohol was added to the tube followed by distilled water. The contents in the tube were thoroughly mixed till no white particles were seen. The tubes were then centrifuged at 1100 ×g for 5 minutes and placed in water bath at 65°C with the stopper downwards from 2 to 3 minutes. The fat column separated was read and taken as percentage of fat in the samples.

### 2.9. Salt Content (NaCl)

The salt content in cheese was determined according to a method described by Breene and Price [18]. Ten grams sample was weighed in a conical flask, and then 50 mL of warm distilled water (50–60°C) was added and stirred until a homogenous suspension was obtained. The suspension was transferred quantitatively to a 250 mL graduated cylinder, diluted to 250 mL with distilled water, mixed thoroughly, and allowed to stand for 5–10 min till the suspended cheese settled out. Thereafter, an aliquot (25 mL) of the supernatant was withdrew and mixed with 2 mL of 2% potassium chromate. The mixture was then titrated with 0.018 N solution of silver nitrate (2.906 g/L). The first discernible color change due to the red color of precipitated silver chromate was taken to be the end point. Knowing that each mL of silver nitrate solution is equivalent to 0.1% salt, the % salt was then calculated.

### 2.10. Formol and Shilovish Ripening Indices

The method of Abdel-Tawab and Hofi [19] was used to measure the Formol and Shilovish Ripening Indices. These titration indices are used to assess the degree of hydrolysis during cheese ripening by measuring the total *α*-amino nitrogen concentration of free amino acids, oligopeptides, and polypeptides in protein hydrolysates. These assays are based on the fact that the reaction of the cheese sample with formaldehyde releases a proton (H^+^) from the free amino group that can be titrated with an alkaline solution. Thereafter, the amount of alkali added is considered to be equivalent to the amount of *α*-amino groups present in the protein solution. In this method, 5 grams of cheese was weighed, emulsified with warm distilled water (50–60°C), made up to 55 mL with distilled water at 66°C, and then filtered (Whatman 42 filter paper). Ten mL of the filtrate was titrated with 0.1 N NaOH using 0.5 mL phenolphthalein indicator (Titre A). Then, 2 mL of a neutralized formaldehyde solution was added to the flask contents and titration was continued with 0.1 N NaOH to a second end point (Titre B). Thereafter, a second titration for the original was done again (10 mL) with 0.1 N NaOH using phenolphthalein indicator (10 drops 0.1 g/100 mL of 50% ethyl alcohol) till a blue color was reached and the volume of alkali used for this titration was recorded (Titre C). The ripening indices of cheese were calculated as follows:

(1) $$\begin{array}{c} \text{Formol}\;\text{Ripening}\;\text{Index}\;\left(\text{FRI}\right)=\left(\text{Titre}\;\text{A}-\text{Titre}\;\text{B}\right)\times 100,\\ \text{Shilovish}\;\text{Ripening}\;\text{Index}\;\left(\text{SRI}\right)=\text{Titre}\;\text{C}\times 100. \end{array}$$

### 2.11. Statistical Analysis

The data collected were subjected to analysis of variance (ANOVA) according to Steel et al. [20] and whenever appropriate Duncan multiple range test (DMRT) was used to separate the means. Statistical analysis for science package was used to perform the general linear model at a probability level of 0.05 [21].

## 3. Results and Discussion

Effect of heat treatment, salt concentration, and storage periods on physicochemical properties of braided cheese is shown in Table 1. The pH of BCRM and BCPM decreased (*P* ≤ 0.05) as storage periods progressed and salt concentration increased. At the end of storage period, the pH of BCRM (2.70) stored in unsalted whey was lower (*P* ≤ 0.05) than that of BCPM (3.23). Throughout the storage periods, the pH of BCRM ripened in 5% and 10% SW decreased faster than that of BCPM. A similar observation on the pH of braided cheese was noted earlier [15]. The TA of BCRM and BCPM increased (*P* ≤ 0.05) with the increase in salt concentration and storage periods. The TA of BCRM ripened for 90 days in unsalted whey was higher (*P* ≤ 0.05) than that of BCPM. Abd El Razig et al. [15] and Abd El-Wahab [22] reported that the TA of braided cheese ripened in 10% SW increased gradually in the first day from 0.43% to 2.10% after 2 months of storage. The moisture content of BCRM and BCPM increased (*P* ≤ 0.05) as storage periods progressed and salt concentration increased. The moisture content of BCRM in unsalted whey increased from 44.83% to 56.77% at 90 days of storage, whereas that of BCPM increased to 59.03%. After 60 days of storage, the BCPM stored in 5% and 10% SW showed the highest increase in moisture content to 66.86% and 68.51%, respectively. The BCPM ripened in 10% SW had the highest moisture percent than the other cheese treatment at the end of storage periods. These results agree with those of Bakheit [23] who reported that moisture content of muddaffara cheese gradually increased from 44.6 ± 0.70% at day 0 to 66 ± 0.33% at 8 weeks of storage at 7 ± 2°C. The total solids content of braided cheese as affected by heat treatment was significantly (*P* ≤ 0.05) decreased as storage periods and salt concentration increased. The total solids content decreased in both cheese types throughout the storage periods. In unsalted whey, the total solids content of BCRM decreased from 55.17% to 43.23% and that of BCPM decreased from 54.87% to 40.97% at 90 days of storage. BCPM ripened in 5% and 10% SW showed fast decline in total solids content compared to the BCRM after 90 days of storage. Abd El-Wahab [22] reported that the change in total solids of braided cheese sample stored at cold temperature decreased from 60.06% in the first day to 52.2% after 2 months of storage. The decrease in total solids of the cheese was assumed to be due to either increase in moisture content and dissolution of protein, fat, and salt into pickling solution or the absorption of pickling whey by curd [24]. On the other hand, the protein content of BCRM and BCPM decreased (*P* ≤ 0.05) as the storage period and salt concentration levels increased. At the end of storage period, the protein content of BCRM ripened in 0% salt whey decreased from 27.62% to 20.96%, whereas that of BCPM in unsalted whey decreased from 27.39% to 15.24%. The BCPM ripened in 5% and 10% SW showed higher decrease of protein content 11.25% and 10.12%, respectively, than BCRM ripened for 90 days at the same level of salt concentration. These results agree with the findings of Abd El-Wahab [22] and Bakheit [23]. The SN content of BCRM and BCPM increased (*P* ≤ 0.05) as storage period and salt concentration increased. At the end of storage periods, the SN content of BCRM and BCPM ripened in 0% SW increased (*P* ≤ 0.05) from 0.95% to 3.81% and from 0.89% to 2.67%, respectively. At the end of storage, the SN content of BCRM and BCPM in 5% SW increased from 0.89% to 3.69% and 2.85%, respectively, while that in 10% SW was increased from 0.83% and 0.77% to 3.69% and 2.80%, respectively. Also it was noticed that changes in the SN content were more pronounced in the BCRM than in the BCPM. These results are similar to the findings of Bakheit [23].

The fat of BCRM and BCPM decreased (*P* ≤ 0.05) as storage period progressed and salt concentration levels increased. The fat content of BCRM and BCPM in unsalted whey decreased from 23.0% to 17.0% and 14.0%, respectively, at the end of storage periods (Table 1). Abdalla et al. [25] reported a fat content in the range of 22.22–28.61% for Sudanese muddaffara cheese collected from Khartoum markets. At the end of storage period, the BCPM in 5% and 10% SW showed faster decrease of fat content to 9.33% and 8.33%, respectively, than BCRM. Kim et al. [26] provided evidence that cheddar cheese stored at cold and room temperature and ripened in whey showed a decrease in fat content. Generally at any one storage period or SW concentration, the salt content of BCRM was always lower than that of BCPM (Table 1). The salt content of the braided cheese increased with the increases of the salt concentration of the whey in which the cheese was ripened and with the increase of the storage period. Abd El Razig et al. [15] reported that the salt content of low salt (10%), at the end of storage periods (60 days), was significantly (*P* ≤ 0.05) lower than both medium level (15%) and high level (20%) of salted cheese. Moreover, Salama et al. [27] reported that, during storage, the higher the concentration of salt in pickling brine, the higher the salt content of final cheese product. The FRI and SRI of braided cheese as affected by heat and salt concentration were significantly (*P* ≤ 0.05) increased as storage periods progressed and salt level were elevated. The FRI of BCRM stored in unsalted whey increased from 26.67 in day 0 to 96.67 at 90 days of storage compared to that of BCPM under the same conditions. The FRI of BCRM and BCPM stored in 5% and 10% salted whey was increased substantially during the storage period. These results agree with those of Saleem et al. [28] who found that changes in FRI of white pickled soft cheese followed the same trend as SN content increased gradually with storage and is slightly higher in braided cheese stored in low salted whey. The SRI of BCRM in 0% SW increased from 53.33 at day 0 to 143.33 at day 90 of storage, whereas SRI of BCPM in 0% SW was 146.67 at day 90 of storage. The SRI of BCRM ripened in 5% and 10% SW increased to 163.33 and 150.00 at day 90 of storage, respectively. At the end of storage, the SRI of BCPM stored in 5% and 10% SW increased to 140.00 and 136.67, respectively. El-Gazzar and El-Sayed [29] found that SRI was generally higher in cheese stored in 10% salt than in cheese stored in 15% salt. In the current study, both ripening indices, that is, FRI and SRI, followed a similar trend to that of SN indicating a possible positive association.

## 4. Conclusion

Substantial portion of Sudanese braided cheese is produced in rural areas from raw milk. Such a practice necessitated conduction of this piece of research, particularly braided cheese consumption in Sudan, is hiking. The minor components of BCRM or BCPM such as TA, SN, and salt content increased significantly, whereas their major components such as total solid, protein, and fat contents decreased (*P* ≤ 0.05) as storage period and the salt level of the whey in which the cheese was ripened increased. Both FRI and SRI of the braided cheese increased with the increase in storage period and the salt level of the whey in which the cheese was ripened; however, the change was more pronounced in BCRM than in BCPM. It is recommended to ripen braided cheese in whey with low salt concentration as well as avoid ripening cheese in high salted whey for a prolonged period to minimize reduction in protein content. Further studies should specifically focus on the microbiological aspects of braided cheese made from raw milk and ripened in low salt whey.

## Figures and Tables

**(a):** 

Heat treatment	Salt concentration (%)	Storage period (days)	Parameters				
			pH	TA (%)	Moisture (%)	TS (%)	Protein (%)
BCRM	0	0	5.42^a^	0.48^i^	44.83^c^	55.17^a^	27.62^a^
		30	3.27^g^	0.97^k^	50.89^b^	49.11^ab^	23.46^b^
		60	3.27^g^	1.99^b^	53.07^b^	46.93^b^	22.27^b^
		90	2.70^h^	2.47^a^	56.77^b^	43.23^b^	20.96^b^
	5	0	5.27^b^	0.45^i^	44.08^c^	55.92^a^	28.10^a^
		30	3.64^gh^	0.58^h^	51.10^b^	48.90^b^	23.75^b^
		60	3.60^gh^	1.25^f^	55.30^b^	44.70^b^	21.43^b^
		90	3.20^g^	1.67^c^	60.12^b^	39.88^b^	17.62^c^
	10	0	5.47^a^	0.43^i^	45.32^bc^	54.68^a^	27.98^a^
		30	4.39^c^	0.58^h^	53.97^b^	46.03^b^	22.14^b^
		60	3.90^cdf^	0.86^g^	58.33^b^	41.67^b^	21.55^b^
		90	3.67^f^	1.37^f^	62.58^b^	37.42^c^	16.67^c^

BCPM	0	0	4.96^ab^	0.50^i^	51.13^bc^	54.87^a^	27.39^a^
		30	4.41^c^	0.63^h^	51.90^b^	48.10^b^	21.91^b^
		60	3.39^gf^	0.75^g^	56.50^b^	43.50^b^	21.67^b^
		90	3.23^f^	1.67^c^	59.03^b^	40.97^b^	15.24^c^
	5	0	5.47^a^	0.42^i^	46.91^bc^	54.42^a^	27.74^a^
		30	4.82^b^	0.73^g^	54.79^b^	45.21^b^	22.14^b^
		60	4.04^d^	0.64^h^	66.86^ab^	33.14^c^	12.38^c^
		90	4.18^c^	1.37^f^	68.30^a^	31.70^c^	11.25^c^
	10	0	5.50^a^	0.45^i^	45.82^c^	54.18^a^	26.43^a^
		30	4.82^b^	0.59^hi^	57.00^b^	43.00^b^	20.72^b^
		60	4.50^c^	0.65^h^	68.51^a^	31.49^c^	11.19^c^
		90	4.30^c^	1.43^f^	70.81^a^	29.19^c^	10.12^c^

Means (*n* = 3) followed by different superscripts letters in each column are significantly different (*P* ≤ 0.05).

BCRM: braided cheese processed from raw milk.

BCPM: braided cheese processed from pasteurized milk.

FRI: Formol Ripening Index and SRI: Shilovish Ripening Index.

**(b):** 

Heat treatment	Salt concentration (%)	Storage period (days)	Parameters				
			Fat (%)	Salt (%)	SN (%)	FRI	SRI
BCRM	0	0	23.00^a^	0.57^d^	0.95^c^	26.67^c^	53.33^c^
		30	20.33^a^	0.45^d^	2.08^b^	46.67^c^	103.33^b^
		60	17.67^b^	1.37^d^	2.73^b^	70.00^bc^	136.67^ab^
		90	17.00^b^	2.17^c^	3.81^a^	96.67^ab^	143.33^a^
	5	0	23.00^a^	2.60^c^	0.89^c^	26.67^c^	53.33^c^
		30	18.67^b^	2.97^ c^	2.32^b^	56.67^bc^	106.67^a^
		60	16.6^bc^	3.63^c^	2.44^b^	76.67^c^	133.33^a^
		90	14.67^c^	5.37^a^	3.69^a^	106.67^a^	163.33^a^
	10	0	22.00^a^	2.90^c^	0.83^c^	26.67^c^	53.33^c^
		30	18.00^b^	3.35^c^	2.02^b^	56.67^bc^	100.00^b^
		60	16.00^c^	4.80^b^	2.55^b^	76.67^b^	123.33^b^
		90	14.00^c^	6.27^a^	3.69^a^	100.0^a^	150.00^a^

BCPM	0	0	23.00^a^	2.27^c^	0.89^c^	26.67^c^	53.33^c^
		30	17.67^b^	2.60^c^	1.42^bc^	50.00^c^	100.00^b^
		60	17.67^b^	2.41^c^	2.08^b^	76.67^b^	120.00^b^
		90	14.00^c^	3.33^c^	2.67^b^	93.33^a^	146.67^a^
	5	0	22.67^a^	4.30^b^	0.89^c^	26.67^c^	53.33^c^
		30	16.00^c^	5.07^a^	1.31^c^	46.67^c^	101.67^ab^
		60	11.00^d^	6.13^a^	2.32^b^	63.33^bc^	113.33^ab^
		90	9.33^d^	6.63^a^	2.85^b^	85.00^ab^	140.00^a^
	10	0	22.33^a^	5.00^ab^	0.77^c^	26.67^c^	53.33^c^
		30	15.33^bc^	5.42^a^	1.13^c^	43.33^c^	93.33^bc^
		60	10.67^d^	7.17^a^	1.90^bc^	60.00^c^	110.00^ab^
		90	8.33^d^	7.13^a^	2.80^b^	80.00^ab^	136.67^ab^

Means (*n* = 3) followed by different superscripts letters in each column are significantly different (*P* ≤ 0.05).

BCRM: braided cheese processed from raw milk.

BCPM: braided cheese processed from pasteurized milk.

SN: soluble nitrogen, FRI: Formol Ripening Index, and SRI: Shilovish Ripening Index.
